# Precision medicine‐based therapies in advanced colorectal cancer: The University of California San Diego Molecular Tumor Board experience

**DOI:** 10.1002/1878-0261.13202

**Published:** 2022-04-08

**Authors:** Bryan H. Louie, Shumei Kato, Ki Hwan Kim, Hyo Jeong Lim, Suzanna Lee, Ryosuke Okamura, Paul T. Fanta, Razelle Kurzrock

**Affiliations:** ^1^ Center for Personalized Cancer Therapy and Division of Hematology and Oncology Department of Medicine UC San Diego Moores Cancer Center La Jolla CA USA; ^2^ Division of Hematology and Medical Oncology Department of Internal Medicine Seoul National University Boramae Medical Center Seoul Republic of Korea; ^3^ Department of Internal Medicine Veterans Health Service Medical Center Seoul Republic of Korea

**Keywords:** colorectal cancer, combinatorial treatment, Molecular Tumor Board, N‐of‐One, precision oncology, tumor alterations

## Abstract

Treatment for advanced colorectal cancer is often limited by complex molecular profiles, which promote resistance to systemic agents and targeted monotherapies. Recent studies suggest that a personalized, combinatorial approach of matching drugs to tumor alterations may be more effective. We implemented a precision medicine strategy by forming a Molecular Tumor Board (MTB), a multidisciplinary team of clinicians, scientists, bioinformaticians and geneticists. The MTB integrated molecular profiling information and patient characteristics to develop N‐of‐One treatments for 51 patients with advanced colorectal cancer. All patients had metastatic disease and 63% had received ≥ 3 prior therapy lines. Overall, 34/51 patients (67%) were matched to ≥ 1 drug recommended by the MTB based on individual tumor characteristics, whereas 17/51 (33%) patients received unmatched therapies. Patients who received matched therapy demonstrated significantly longer progression‐free survival (hazard ratio [HR], 0.41; 95% confidence interval [CI], 0.21–0.81; *P* = 0.01) and a trend towards higher clinical benefit rates (41% vs. 18%, *P* = 0.058) (all multivariate) compared to patients receiving unmatched therapy. The MTB facilitated personalized matching of drugs to tumor characteristics, which was associated with improved progression‐free survival in patients with advanced colorectal cancer.

## Graphical abstract

Treatment of advanced colorectal cancer is limited by complex molecular profiles, which promote resistance to systemic agents. We implemented a multidisciplinary Molecular Tumor Board to facilitate matching of drugs to molecular alterations and develop N‐of‐One treatments. Here, we show that this precision medicine strategy is associated with improved clinical outcomes in 51 patients with advanced colorectal cancer.

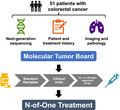

**Abbreviations**
cfDNA: cell‐free DNA
CI: confidence interval
CR: complete response
CRC: colorectal cancer
HR: hazard ratio
MSI: microsatellite instability
MTB: Molecular Tumor Board
NGS: next‐generation sequencing
OS: overall survival
PFS: progression‐free survival
PR: partial response
SD: stable disease
TMB: tumor mutational burden

## 1 Introduction

Colorectal cancer (CRC) is the third most commonly diagnosed cancer worldwide and the second most frequent cause of cancer‐related death, with approximately 1.8 million new cases diagnosed each year and approximately 900 000 deaths annually [1]. Surgery and chemotherapy have long been the backbone of CRC treatment [2]. Standard chemotherapy regimens commonly include 5‐fluorouracil, oxaliplatin, irinotecan, and capecitabine in various combinations [2]. Furthermore, certain targeted therapies have become regularly used in CRC treatment. Anti‐EGFR agents (cetuximab, panitumumab) are approved for first‐line therapy in metastatic CRC, with selection for *RAS* wild‐type patients [3]. Anti‐VEGF/VEGFR agents such as bevacizumab are approved for first‐ and second‐line treatment of CRC, without biomarker selection [2]. Unfortunately, the 5‐year relative survival rate is still only ~ 14% for patients with advanced CRC [4].

Recently, the deployment of next‐generation sequencing (NGS) has led to the development of other targeted approaches that aim to directly match drugs with molecular alterations [5, 6, 7, 8]. One example is the NTRK inhibitor larotrectinib, which has shown an overall response rate of ~ 75% in solid tumors with *NTRK* fusions [9]. Another example is the utilization of anti‐PD‐1 immunotherapies (pembrolizumab, nivolumab), which are approved for metastatic CRC with mismatch repair (MMR) deficiency and/or high microsatellite instability (MSI‐H), showing a ~ 55% overall response rate in these patients [10, 11, 12]. Ultimately, while the strategy of selecting patients for therapy based on genomic alterations has shown promise in many settings, the majority of patients with CRC who undergo single‐agent targeted therapy demonstrate limited responses, and rapidly develop therapeutic resistance [2].

The effectiveness of matched targeted monotherapy is often limited by the genomic heterogeneity that exists between tumors of the same tissue type and the fact that most advanced cancers have molecular profiles that are complex [13]. Even when targetable molecular alterations are identified, it is difficult to determine their driver vs. passenger status in tumors with multiple genomic aberrations [6, 14, 15, 16, 17]. Recent studies suggest that the optimal strategy may involve a combinatorial use of therapies in order to maximize the matching of drugs to molecular alterations [18, 19].

We implemented a clinical precision medicine strategy facilitated by a Molecular Tumor Board (MTB) [20, 21, 22, 23]. This diverse, multidisciplinary team of clinicians and scientists functions by incorporating a comprehensive review of each patient’s unique characteristics, including molecular profiling, imaging, pathology, laboratory findings, and clinical history, in order to develop an N‐of‐One treatment plan discussed for each cancer patient. The idea of the MTB has been a growing treatment paradigm in the field of precision oncology with variable success in clinical practice [21, 22, 24, 25]. For example, some MTBs have demonstrated objective response rates as high as 44–67% in patients with non‐small cell lung cancer [26, 27]. However, other MTBs treating various solid and hematologic cancers have shown objective response rates from 0–13% [28, 29]. In the case of CRC, the use of an MTB to match molecularly targeted regimens to patients has recently shown improved outcomes in patients with metastatic CRC [30]. Here, we present 87 patients with advanced colorectal cancer who were presented to the University of California San Diego, Center for Personalized Cancer Therapy Molecular Tumor Board and demonstrate several lines of evidence to support that recommended matched therapies are associated with improved clinical outcomes.

## 2 Patients and methods

### 2.1 Molecular Tumor Board

The meetings for the face‐to‐face Molecular Tumor Board (MTB) discussions consisted of one to one and a half hour sessions, approximately three times per month, covering the cases submitted by treating physicians. An MTB project manager organized the meeting agendas including de‐identified patient information (age, sex, attending physician, diagnosis, treatment history, pathology) and the molecular profiling information, including test used and molecular diagnostics report as well as the date of specimen. In addition, the project manager assisted with ordering tests from certified laboratories and obtaining consent when needed.

The MTB meetings were led by a senior and mid‐level medical oncologist with comprehensive experience in genomics, clinical trials, medical oncology and immunotherapy. The MTB consisted of a wide range of specialists including medical, radiation, and surgical oncologists as well as radiologists, pathologists, geneticists, clinical trial coordinators, translational/basic science researchers, and bioinformaticians. The pathology, imaging, clinical history, and laboratory tests were evaluated. All laboratory tests were Clinical Laboratory Improvement Amendments (CLIA)‐licensed and College of American Pathologist (CAP)‐accredited. Furthermore, discussion focused on the molecular profiling of each patient, assessing the impact of known alterations on cancer pathways, the possibility of germline mutations, and whether there were drugs, either in clinical trials or FDA approved, which could target the aberrations present. Medication acquisition specialists and clinical trial coordinators/navigators present at the MTB enabled obtaining medications (either on‐ or off‐label approved) and screening for available clinical trials. Throughout all MTB discussions, all HIPAA privacy laws were closely followed. The accuracy of the MTB report was thoroughly reviewed by the presenting physician and the MTB moderator before it was written into the medical record. MTB recommendations were considered advisory, with all treatment decisions to be made by the responsible physician.

### 2.2 Patients and therapy

**Fig. 1:**
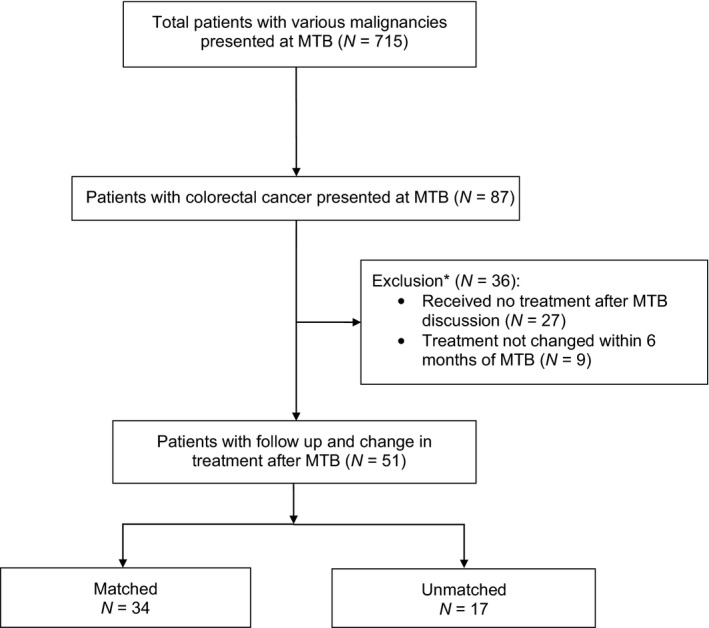
Consort diagram of 87 colorectal cancer patients presented at face‐to‐face Molecular Tumor Board (MTB) [23]. *Excluded patients were most often those who presented to the MTB for assessment of future treatment strategies, but without need for immediate treatment.

This study evaluated 87 patients with CRC out of the total 715 patients with various malignancies who presented face‐to‐face at the Molecular Tumor Board (MTB) between December 2012 and September 2018 (Fig. 1) [23]. Among these patients, those who did not receive treatment following MTB discussion (*N* = 27) or those whose treatment did not change within 6 months following MTB discussion (*N* = 9) were excluded from the cohort. These patients were excluded most often because they presented to the MTB to assess potential future treatment strategies but did not need immediate treatment (Patients who received treatment more than 6 months after the MTB were excluded from this report). The remaining 51 patients with colorectal cancer whose treatment changed following MTB, and their subsequent clinical follow up were included for analysis. All patients who had a change in therapy had shown tumor progression or intolerance to the prior therapy. Patients may have been treated with approved drugs (on‐ or off‐label) or with investigational drugs (on a secondary clinical trial). The physician chose the treatment and could follow or not follow the MTB recommendations. Electronic medical records were reviewed for de‐identified characteristics and outcome data. This present study adhered to the guidelines established by the Internal Review Board (IRB)‐approved University of California San Diego (UCSD) Profile Related Evidence Determining Individualized Cancer Therapy (PREDICT) study (NCT02478931) and any additional investigational studies for which patients gave written and informed consent. The study methodologies also conformed to the standards set by the Declaration of Helsinki.

### 2.3 NGS of tissue and cell‐free circulating tumor DNA (cfDNA)

Tissue and blood‐derived cfDNA NGS were obtained from one of several Clinical Laboratory Improvement Amendment (CLIA) certified laboratories: Founda‐tion Medicine (https://www.foundationmedicine.com/), Tempus (https://www.tempus.com/genomic‐profiling/), Guardant (https://guardanthealth.com/), University of California San Diego Health (https://health.ucsd.edu/), Memorial Sloan Kettering Cancer Center (https://www.mskcc.org/). Tissue panels ranged from 182 to 596 genes and blood‐derived cfDNA panels ranged from 54 to 74 genes. In particular patients, protein expression and mRNA analysis were also conducted, as well as evaluation of specific immune markers. The treating physician decided tests ordered. Variants of unknown significance were not considered in therapy decisions.

### 2.4 Statistical methods and endpoints

The patients and their molecular characteristics were presented with descriptive statistics. Outcome variables included progression‐free survival (PFS) and overall survival (OS). PFS was defined as the time between the treatment start date after MTB presentation and the date of progression, determined by clinical findings or imaging. OS was defined as the length of time from the treatment start date after MTB presentation and the last follow‐up date. Patients were censored for PFS at last follow‐up date if they had ongoing therapy without progression at that date. Patients were censored for OS if they were alive at last follow‐up date. Clinical response was evaluated based on RECIST criteria [31]. For survival analysis, Kaplan–Meier analysis with Cox regression was used to compare subgroups of patients. Patients without progression (for PFS) or still alive (for OS) at the time of last follow up were censored at that date. To compare clinical benefit rates, binomial logistic regression was used. *P*‐values ≤ 0.05 were considered significant. Statistical analyses were performed with R and SPSS, version 25 (IBM Corporation, Armonk, NY, USA).

### 2.5 Matching

Treatment was considered “matched” if ≥ 1 compound in the therapy regimen targeted ≥ 1 aberration or pathway component aberrant in a patient's molecular profile or a functionally active protein preferentially expressed in the tumor with an IC_50_ value in the low nmol·L^−1^ range (for small molecule inhibitors) or if the aberration was the primary target for antibodies. *BRCA* mutations and other alterations that cause homologous DNA repair defects were considered matched to PARP inhibitors or platinum agents. Checkpoint blockade was considered matched if the patient had intermediate or high tumor mutation burden (TMB), positive immunohistochemistry for PDL1 or specific tumor alterations such as *PDL1* amplification. Detailed information on the matched patients, their molecular characteristics and the specific drugs that were suggested by the MTB is provided in Table S1. Additionally, specific alterations and rationale for the matched therapies that were given are provided in Table S2. Further details on the MTB matching have also been previously reported [18, 23].

## 3 Results

### 3.1 Patient characteristics

Of the total 87 patients with CRC that presented in the face‐to‐face MTBs, 51 individuals were assessable for therapeutic clinical outcome (Table 1). The most common reason that patients were inevaluable was that they were presented by the physician at MTB before progression in order to hear about back‐up future plans, but the patient then did not require therapy for at least 6 months. Among these 51 patients, the median age was 56 years (range: 31–74). Twenty‐seven patients (53%) were women, and 24 patients (47%) were men. The patients had some form of advanced or metastatic colorectal cancer. Most patients had colorectal adenocarcinoma; however, two patients had rectal squamous cell carcinoma. Lastly, 32 of the patients (63%) had ≥ 3 therapies prior to MTB presentation.

**Table 1:** Baseline demographics and sequencing tests of CRC patients presented at the face‐to‐face Molecular Tumor Board (MTB) and assessable for clinical treatment outcome (*N* = 51).

Total patients with colorectal cancer (*N* = 51)	
Period	December 2012–September 2018
Median age at MTB (years) (range)	56 (31–74)
Sex, *N* (%)	Men, 24 (47%); Women, 27 (53%)

Next‐generation sequencing (NGS)	*N*, patients
Tissue‐based sequencing	47
Foundation Medicine	44
UCSD NGS	1
MSKCC NGS	1
Tempus NGS	1
Liquid biopsy	30
Guardant cfDNA	27
Foundation Medicine cfDNA	3

Abbreviations: cfDNA, cell‐free DNA; MTB, Molecular Tumor Board; MSKCC, Memorial Sloan Kettering Cancer Center; UCSD, University of California, San Diego.

### 3.2 Molecular characteristics of patients showed variable and complex molecular portfolios

All molecular profiling reports performed by clinical‐grade laboratories for each patient were evaluated during the MTB discussion. Tissue NGS was performed in 47 patients at four different laboratories and cfDNA analysis was performed in 30 patients at two laboratories (Table 1).

**Fig. 2:**
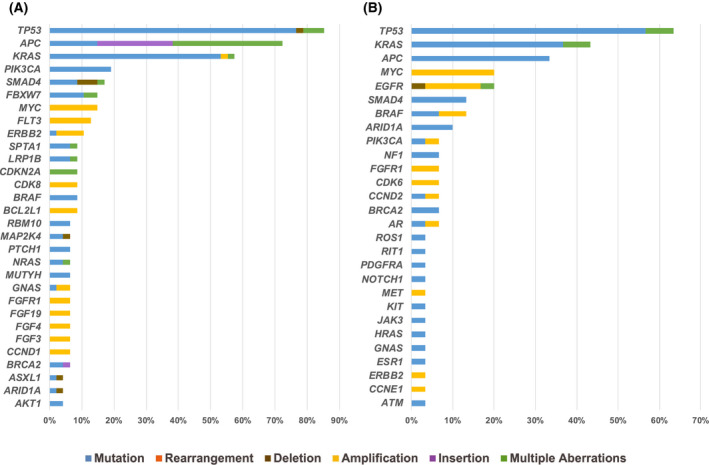
Frequency of characterized genomic alterations from tissue NGS and cfDNA of colorectal cancer. (A) Alterations identified by tissue NGS (*N* = 47). Alterations present in ≥ 4% of patients were included. (B) Alterations identified by cell‐free DNA (*N* = 30). Alterations present in ≥ 3% of patients were included. Colored bars show the percent of patients with the specific type of genomic alteration for each gene. Multiple aberrations indicates that some patients harbored multiple types of alterations (e.g. mutation, deletion, insertion) within the same gene.

From tissue NGS of CRCs (*N* = 47), *TP53* was the most commonly altered gene (85% [40/47]) followed by *APC* (72% [34/47]), *KRAS* (57% [27/47]), *PIK3CA* (19% [9/47]), and *SMAD4* (17% [8/47]) (Fig. 2A). Alterations detected by tissue NGS included mutations, deletions, amplifications, insertions and multiple aberrations of genes.

Among CRCs with blood‐derived cfDNA profiling (*N* = 30), the most commonly altered genes were *TP53* (63% [19/30]) followed by *KRAS* (43% [13/30]), *APC* (33% [10/30]), *MYC* (20% [6/30]), and *EGFR* (20% [6/30]) (Fig. 2B). Alterations detected by cfDNA profiling included mutations, deletions, amplifications and multiple aberrations of genes.

### 3.3 Patients who were matched to therapy had longer PFS than those with unmatched therapy

Among the 51 evaluable colorectal cancer patients, 34 (67%) were matched to ≥ 1 drug recommended by the MTB. The remaining 17 patients received an unmatched therapy following MTB discussion.

**Fig. 3:**
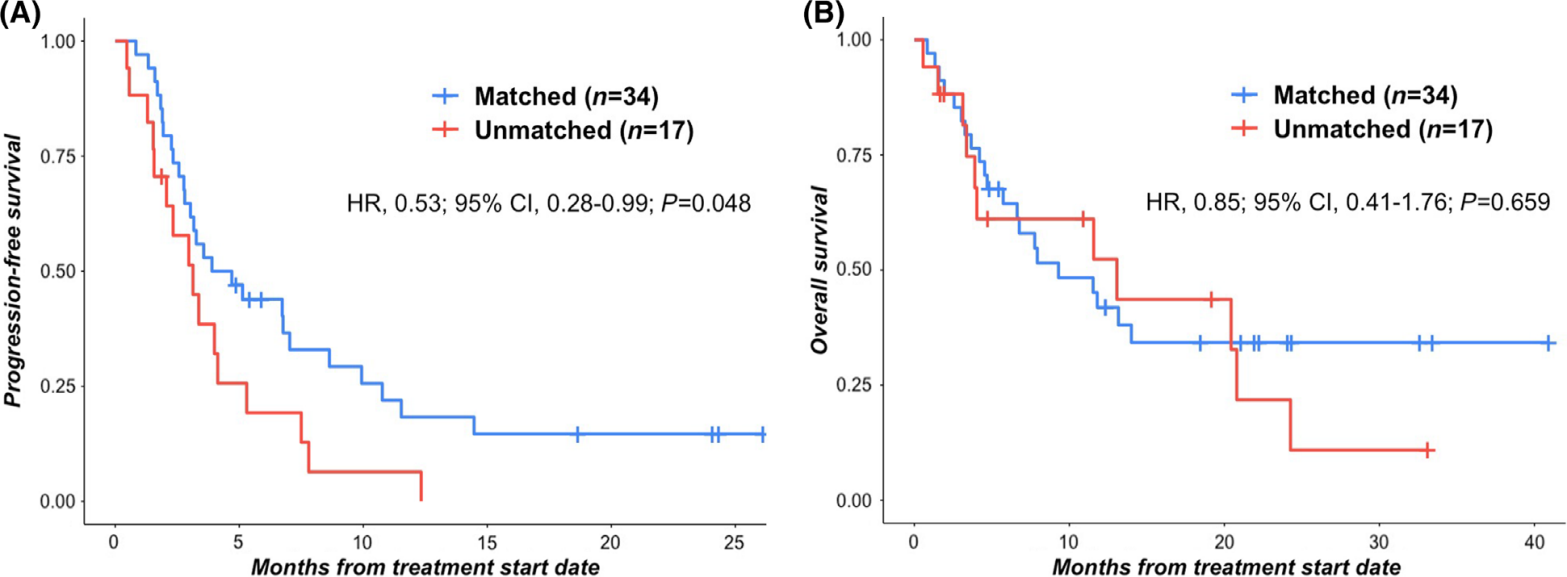
Progression‐free survival and overall survival in matched vs. unmatched patients. (A) Progression‐free survival (PFS) in patients who received matched vs. unmatched therapy (*N* = 51). Median PFS: whole cohort, 3.6 months (95% CI: 2.6–4.6); matched patients, 3.9 months (95% CI: 1.3–6.5); unmatched patients, 3.1 months (95% CI: 1.6–4.7). Hazard ratio (HR) calculated by univariate Cox regression. (B) Overall survival (OS) in patients who received matched vs. unmatched therapy (*N* = 51). Median OS: whole cohort, 11.5 months (95% CI: 6.5–16.5); matched patients, 9.3 months (95% CI: 3.9–14.7); unmatched patients, 13.1 months (95% CI: 0–27). Hazard ratio (HR) calculated by univariate Cox regression. Notably, of the 17 unmatched patients, 4 patients died upon progression of disease from initial treatment (i.e., date of progression equals the date of death). Of the remaining 13 unmatched patients, 8 (62%) received matched targeted therapy following progression (potentially confounding the OS).

Patients who received matched therapy had significantly improved PFS when compared to patients who received unmatched therapy (HR, 0.53; 95% CI, 0.28–0.99; *P* = 0.048 [univariate analysis]) (Fig. 3A). The association between matched patients and improved PFS remained significant after multivariate analysis (HR, 0.41; 95% CI, 0.21–0.81; *P* = 0.01) (Table 2). In contrast, matched patients exhibited no significant improvement in OS when compared to unmatched patients (HR, 0.85; 95% CI, 0.41–1.76; *P* = 0.659 [univariate analysis]) (Fig. 3B). Notably, of the 17 unmatched patients, four patients died upon progression of disease from initial treatment (i.e., date of progression equals the date of death). However, 8 (62%) of the remaining 13 patients whose disease progressed on their initial unmatched therapy, were subsequently switched by their treating physician to the matched targeted therapy that was originally recommended by the MTB (potentially confounding the OS).

**Table 2:** Association between patient and treatment characteristics and PFS (*N* = 51).

Characteristics		PFS				
	*N*	Median (months) (95% CI)	Univariate		Multivariate^a^	
			HR (95% CI)	*P*‐value	HR (95% CI)	*P*‐value
Age, years						
≥ 56	25	3.57 (2.15–4.98)	0.76 (0.42–1.38)	0.368	–	–
< 56	26	3.17 (0.65–5.68)	Reference	–	–	–
Sex						
Men	24	3.17 (2.70–3.63)	1.09 (0.59–2.00)	0.782	–	–
Women	27	3.90 (1.64–6.16)	Reference	–	–	–
Matched treatment						
Yes	34	3.90 (1.28–6.52)	0.53 (0.28–0.99)	0.048	0.41 (0.21–0.81)	0.01
No	17	3.13 (1.62–4.65)	Reference	–	Reference	–
Number of prior lines of therapy						
≥ 3	32	3.17 (2.71–3.63)	1.40 (0.75–2.61)	0.298	–	–
< 3	19	6.77 (0.89–12.64)	Reference	–	–	–
Received chemotherapy^b^						
Yes	24	5.30 (2.28–8.32)	0.58 (0.32–1.07)	0.083	0.46 (0.24–0.88)	0.019
No	27	2.80 (1.70–3.90)	Reference	–	Reference	–

Abbreviations: CI, confidence interval; HR, hazard ratio; PFS, progression‐free survival.

a Covariates with *P* < 0.2 were included in multivariate analysis.

b Patients were categorized as having received chemotherapy if their treatment regimen included any of the following drugs: 5FU, oxaliplatin, irinotecan or capecitabine.

**Fig. 4:**
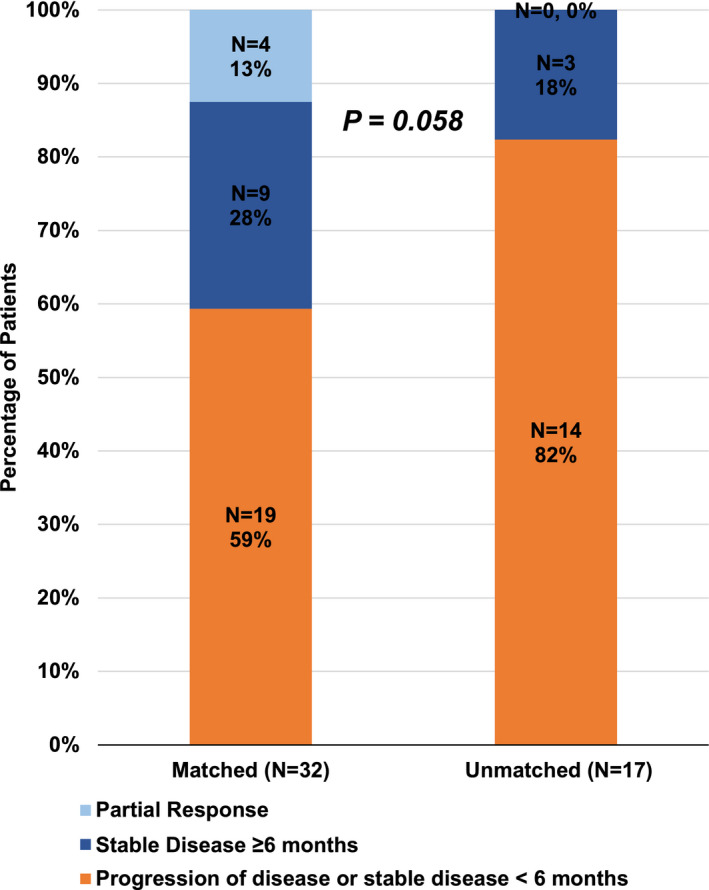
Clinical benefit rate (SD ≥ 6 months/PR/CR) in matched vs. unmatched patients. Clinical benefit rate (SD ≥ 6 months/PR/CR) in patients who received matched (13/32 (41%)) vs. unmatched (3/17 (18%)) therapy (*N* = 49*) (*P* = 0.058, multivariate analysis). *Two patients were not included in this analysis because they had ongoing stable disease that was < 6 months at last follow up and hence it was too early for evaluation of this parameter.

Similar to previous studies, we stratified patients who exhibited stable disease (SD) ≥ 6 months, partial response (PR), or complete response (CR), based on RECIST criteria, as having clinical benefit (SD ≥ 6 months/PR/CR) from treatment, whereas patients who had progressive disease (PD), or stable disease < 6 months, were categorized as not having clinical benefit [23, 31]. Subsequently, patients who received matched therapies showed a trend towards a higher rate of clinical benefit (41% [13/32]) when compared to patients who received unmatched therapies (18% [3/17]) (odds ratio [OR], 0.21; 95% CI, 0.04–1.06; *P* = 0.058; multivariate analysis; Table 3 and Fig. 4).

**Table 3:** Association between patient and treatment characteristics and clinical benefit rate (SD ≥ 6 months/PR/CR) (*N* = 49^a^).

Characteristics		Clinical benefit rate (SD ≥ 6 months/PR/CR)				
	*N*	SD ≥ 6 months/PR/CR, *N* (%)	Univariate		Multivariate^b^	
			OR (95% CI)	*P*‐value	OR (95% CI)	*P*‐value
Age, years						
≥ 56	24	8 (33.3%)	Reference	–	–	–
< 56	25	8 (32.0%)	0.94 (0.29–3.11)	0.921	–	–
Sex						
Men	22	6 (27.3%)	Reference	–	–	–
Women	27	10 (37.0%)	1.57 (0.46–5.31)	0.470	–	–
Matched treatment						
Yes	32	13 (40.6%)	Reference	–	Reference	–
No	17	3 (17.6%)	0.31 (0.08–1.31)	0.112	0.21 (0.04–1.06)	0.058
Number of prior lines of therapy						
≥ 3	30	7 (23.3%)	Reference	–	Reference	–
< 3	19	9 (47.4%)	2.96 (0.86–10.17)	0.085	3.04 (0.75–12.39)	0.121
Received chemotherapy^c^						
Yes	24	12 (50.0%)	Reference	–	Reference	–
No	25	4 (16.0%)	0.19 (0.05–0.72)	0.015	0.16 (0.04–0.71)	0.015

Abbreviations: CI, confidence interval; CR, complete response; OR, odds ratio; PR, partial response; SD, stable disease.

a Two patients were not included in this analysis because they had ongoing stable disease that was < 6 months at last follow up and hence it was too early for evaluation of this parameter.

b Covariates with *P* < 0.2 were included in multivariate analysis.

c Patients were categorized as having received chemotherapy if their treatment regimen included any of the following drugs: 5FU, oxaliplatin, irinotecan or capecitabine.

## 4 Discussion

The MTB experience at the University of California, San Diego Moores Cancer Center demonstrated the use of molecular profiling technologies to characterize and treat advanced CRC. Multiple clinical‐grade testing modalities, including tissue NGS, blood‐derived cfDNA, mRNA and IHC were evaluated and facilitated the MTB discussion. Molecular profiling of 51 patients with metastatic CRC revealed genomic alterations similar in type and frequency to previous reports of common alterations in CRC (Fig. 2) [5, 6, 7, 8]. Ultimately, 34 (67%) of 51 patients were matched and treated with ≥ 1 drug recommended by the MTB, while the remaining 17 (33%) patients were treated with unmatched therapy.

Overall, matched patients had significantly longer PFS when compared to unmatched patients. Furthermore, in multivariate analysis, matched therapy was independently associated with improved PFS (*P* = 0.01) and a trend towards improved clinical benefit rate (SD ≥ 6 months/PR/CR; *P* = 0.058; Tables 2 and 3). However, when comparing OS, there was no significant difference between the matched and unmatched groups. Importantly, upon further analysis, we observed that 8/13 (62%) unmatched patients who received treatment following progression on their unmatched treatment regimen, in fact received matched therapy for a subsequent therapy line. In this case, the treating physician initially decided to use an unmatched therapy, but upon disease progression, opted to change treatment to the matched therapy that was originally recommended by the MTB. This was allowable because the MTB was considered advisory, and the physicians could choose therapy at any time. Therefore, while these patients are placed in the unmatched group based on their initial treatment, their subsequent treatment change to matched therapy after disease progression may be a confounding variable for OS measurements and may explain the discrepancy observed between PFS and OS.

There were a number of limitations to the current study. First, this study represents a retrospective review of real‐world data from the MTB and is not a randomized controlled trial. Therefore, because this study was not a randomized controlled trial, we cannot eliminate the possibility that higher matched patients had a better prognosis. Second, this study had a limited number of CRC patients derived from a larger cohort of patients with multiple types of cancer who presented to the MTB [23]. Another limitation is that some of the matches proposed by the MTB may have had limited impact in CRC. For example, there is some evidence that PARP inhibitors do not improve outcomes in CRC patients with DNA damage repair defects [32]. While previous reports on our MTB stratified patients based on matching score, a percentage that reflects the degree of molecular matching between drugs and patient characteristics, our current study considered only the dichotomization of matched versus unmatched treatments, mainly due to the very small number of patients who had high matching scores (*N* = 7) [18, 23].

## 5 Conclusions

In summary, the current study demonstrates the utility of the MTB in characterizing and treating advanced CRC by integrating multiple profiling modalities and matching molecular alterations to drugs. Our patients all had advanced disease with 63% (32 of 51) having tumors that progressed on ≥ 3 systemic regimens. Patients could be successfully matched to targeted drugs, consistent with prior studies in CRC or to immunotherapy agents, even in the presence of microsatellite stable disease [33]. Patients treated with matched versus unmatched therapies exhibited significantly longer PFS and showed higher clinical benefit rates. Of interest was that, overall survival was not prolonged in patients who received matched versus unmatched therapies, but this outcome parameter may have been confounded by the sizable subgroup which received a matched therapy following progression on their unmatched regimen. Prospective trials such as Personalized ANtibodies for GastroEsophageal Adenocarcinoma (PANGEA) (NCT02213289) and Colorectal and Liquid Biopsy Molecularly Assigned Therapy (COLOMATE) (NCT03765736) are currently underway to investigate the use of various molecular profiling modalities and treatment matching strategies [34, 35]. In conclusion, our study suggests that a multidisciplinary MTB can be of benefit to patients with refractory metastatic CRC. Future studies of larger groups of patients examined prospectively are warranted.

## Conflict of interest

SK serves as a consultant for Foundation Medicine and receives speaker’s fees from Roche. RK has research funding from Incyte, Genentech, Merck Serono, Pfizer, Sequenom, Foundation Medicine, Guardant Health, Grifols and Konica Minolta, as well as consultant fees from LOXO, X‐Biotech, Actuate Therapeutics, Genentech and NeoMed. She receives speaker fees from Roche and has an equity interest in IDbyDNA and Curematch, Inc, and serves on the Board of CureMetrix and CureMatch. BHL, KHK, HJL, SL, RO and PTF have no competing interests.

## Author contributions

RK and SK designed and directed the study. BHL, SK and RK drafted the manuscript. BHL and SK analyzed and interpreted the data. KHK, HJL, RO and SL collected and compiled the data. SK, PTF, RO, SL and RK were involved in the Molecular Tumor Board. All authors have read and approved the manuscript.

## Ethics approval and consent to participate

This present study adhered to the guidelines established by the Internal Review Board (IRB)‐approved University of California San Diego (UCSD) Profile Related Evidence Determining Individualized Cancer Therapy (PREDICT) study (NCT02478931) and any additional investigational studies for which patients gave written and informed consent.

### Peer review

The peer review history for this article is available at https://publons.com/publon/10.1002/1878‐0261.13202.

## Supporting information


**Table S1**. Clinical characteristics and therapies suggested by the MTB for 34 patients with matched therapy.

**Table S2**. Specific alterations and rationale for targeted therapy given to 34 matched patients.

## Data Availability

The datasets used and/or analyzed during the current study are available from the corresponding author upon reasonable request.
